# How Do Persons with Mild Acquired Cognitive Impairment Use Information and Communication Technology and E-Services? Results from a Swedish National Survey

**DOI:** 10.1371/journal.pone.0159362

**Published:** 2016-07-18

**Authors:** Aboozar Eghdam, Aniko Bartfai, Christian Oldenburg, Sabine Koch

**Affiliations:** 1 Health Informatics Centre (HIC), Department of Learning, Informatics, Management and Ethics (LIME), Karolinska Institutet, Stockholm, Sweden; 2 Division of Rehabilitation Medicine, Department of Clinical Sciences Danderyds Hospital, Karolinska Institutet, Stockholm, Sweden; University of Catania, ITALY

## Abstract

**Introduction:**

Mild acquired cognitive impairment is a term used to describe a sub-group of persons with mild cognitive impairment who are expected to reach a stable cognitive level over time. One tactic that can be considered for further developing treatment for this group is the use of information and communication technology and e-services. The purpose of this study was to investigate the current use of regular e-services and social media by this group as well as their user experiences.

**Methods and Materials:**

Data were collected through a self-administered survey and analyzed using quantitative methods. The questionnaire included questions regarding the participants’ use of and experience with e-services. Categorization of e-services was based on and cross-validated with the International Classification of Functioning, Disability and Health (ICF). To estimate participants’ degree and type of impairment, the Cognitive Failure Questionnaire (CFQ), measuring cognitive difficulties in performing everyday tasks, was added.

**Results:**

In total, 282 persons with acquired brain injury participated in the survey. The participants’ CFQ scores showed that they were suffering from mild to moderate cognitive impairments, most often acquired from traumatic brain injuries (40%). The majority (89%) used e-services in different categories whereof the most popular and essential ones were communication services (59%) and banking (39%) services. Participants with higher total CFQ scores (>58) used more e-services in most of the categories compared to participants with lower scores (<31). Although participants were interested in social media, they were annoyed by advertisements and the Internet speed in general. Some participants reported privacy concerns and addictive behavior. However, they mostly considered e-services to be trustworthy and supportive in different contexts. The usage of electronic devices decreased by age with the exception of electronic tablets that were used by older participants approximately as frequently as by other age groups.

**Conclusions:**

Although persons with mild to moderate acquired brain injury used various e-services that are not customized for them, very few participants used self-care health services (apps) and readers (e-readers). Further studies are needed on utilizing these identified aspects for this group to support them with their chronic condition.

## Introduction

Acquired brain injury (ABI) is one of the leading causes of disability in many developed countries [1]. By the year 2030, stroke and Traumatic Brain Injury (TBI) from traffic collisions, assaults or falls will be the major causes of brain injury worldwide [2]. Every year, over 70,000 people suffer from brain injuries in Sweden [3]. Most of them (80%) are mild, 10% moderate and the remaining 10% have serious/severe brain injuries in addition to a large number of unrecorded minor injuries that are not registered [3].

### Cognitive impairment

Cognitive impairment is a common consequence for a wide range of diseases or injuries to the brain [3–5]. Many areas of cognition can be affected, but the most common ones are deficits in memory, attention and various aspects of executive functions, such as problem solving[6–8]. This is especially true for people with mild cognitive impairment who lack the more commonly known and visible signs of brain injury, such as motor or language impairment. Persons with mild cognitive impairment (MCI) are most often able to work and live independently and their impairment may only be noticeable in situations with demands for higher cognitive load, e.g. during extended stress. The cognitive problems that persons with MCI experience may be particularly hard to identify and understand, both for the persons themselves, for their significant others, employers or for primary health care services [6–9].

To our knowledge, there is still no accepted universal definition or psychometric criteria that can be applied in defining MCI. Most work on the concept has been made in Alzheimer research where Peterson has suggested that MCI is characterized by subjective memory complaints, unusually low scores on objective memory measures, and normal activities of daily living [10]. In this line of research, MCI is seen as a pre-clinical phase in a dementing process. However, as mentioned above, MCI is not limited to progressive diseases in the brain but can be the result of an acquired injury. If MCI is the result of such an injury, the condition does not get progressively worse and the person reaches a stable state. To distinguish this condition from MCI due to progressive diseases, Mild Acquired Cognitive Impairment (MACI) has been introduced as a new term [11].

### Mild acquired cognitive impairment

A common cause for an acquired cognitive impairment is traumatic brain injury (TBI). The prognosis for persons with mild TBIs is generally good and most will resume normal functioning within days or a few weeks[12,13]. However, a sizable minority of these people continue to report cognitive problems, as well as emotional and somatic symptoms for months, even years after their injury [13,14]. In addition to traumatic brain injuries a number of medical conditions, such as stroke, neurological diseases and some treatment side effects, e.g. heart-surgery might cause mild cognitive impairment in a presumably sizeable group of the population in working age, constituting a large human and societal problem [4].

Persons with MACI can have multiple cognitive and/or mild physical disabilities and need access to already limited rehabilitation services to improve their affected skills, that can be alleviated by using Information and communication technology (ICT) [15,16]. Thus, one tactic that can be considered for further developing treatment of MACI is the use of ICT.

### Information and communication technology and e-services

The advancement of ICT offers great potential to help people with chronic conditions. A varying proportion of households and individuals use the Internet worldwide. Today about 1.49 Billion adults visit Facebook at least once a month and about 87% of daily active users do so using mobile/smartphones [17]. About 81% of Europeans use the Internet today through their mobile and personal computers [18]. In addition, mobile internet usage has become popular with the rise of new mobile devices such as smartphones or tablet computers [18]. In Sweden 93% of households have access to the Internet. Furthermore, Sweden has the highest number of individuals (82% in 2012–14) among European countries that interact with public authorities over the Internet and about 63% of them use the Internet for seeking health information [18].

Internet is the main channel to ubiquitously receive and use e-services [19]. Rowley defines e-services as: “deeds, efforts or performances whose delivery is mediated by information technology (including the Web, information kiosks and mobile devices)”[19]. E-services have also been considered as technology-based self-services[20]. For individuals with disabilities, e-services have become almost essential in their daily life by providing a variety of useful services and tools in any context of use [21,22], in order to cope with and alleviate their condition [23]. This suggests that e-tools and services need to be utilized in a wide range of situations, and that they need to be adapted to each person’s individual needs [19,20,24].

Although the last decade has seen a large amount of research on how to use e-services to support people with chronic disease, according to a systematic review of 3894 publications [11], persons with cognitive impairments have received less attention [25]. While using e-services can lead to mental and physical improvements for people with different disabilities, barriers for using relevant services may in fact strengthen the unequal status (digital divide) for people with cognitive impairments in the society compare to other people with chronic incapacities [25,26]. Based on our previous work [11], there is a lack of information about what e-services have been created and evaluated for supporting persons with MACI, i.e. for persons not requiring specially constructed supportive devices such as for example NeuroPage [27]. Findings by Tun et al. suggested that using e-services frequently is significantly associated with better cognitive function, executive control and younger age [28]. However, there is a lack of knowledge about how persons with mild or moderate cognitive impairment use e-services today.

The purpose of this study is to investigate the current use of regular e-services and social media by persons with mild or moderate cognitive impairment and to explore their opinions and experiences.

## Methods and Materials

The principal method of data collection was a postal self-administered (self-completion) questionnaire directed towards persons with ABI about their experiences with e-services and social media. Included in the questionnaire were items regarding cognitive problems and their severity in every-day life.

The approval of the study was obtained from the Regional Ethics Committee (2014/513-31/3 April 9, 2014). The Regional Ethics Committee (Regionala etikprövningsnämnden—http://www.epn.se/) has a task to vet cases within the field of medical science (medicine, pharmacology, odontology, the science of health care and clinical psychology). The secretariats of the regional boards are situated at the different universities in Sweden.

### Setting (participant’s recruitment)

The study was done in collaboration with the Swedish Association of Brain Injury (Hjärnkraft). Families, who sought support from each other in a situation when a beloved one suffered from acquired brain injury, founded Hjärnkraft in 1988. The association has about 1300 members with brain injuries who have valid addresses and can be contacted by paper/regular mail. Bearing in mind the population size and based on sample size calculation for determining the frequency of a factor in a population (Table 1), for this study, more than estimated 99% confidence level (n = 600) were selected randomly (and anonymously to the authors) to receive the paper-based questionnaire and respond voluntarily. Hjärnkraft performed the randomization and distribution process.

**Table 1:** Sample Size for Frequency in a Population (from OpenEpi, Version 3, open source calculator—SSPropor).

**Sample Size for Frequency in a Population**	
**Population size(for finite population correction factor or fpc)(*N*):**	1300
**Hypothesized % frequency of outcome factor in the population (*p*):**	50%+/-5
**Confidence limits as % of 100(absolute +/- %)(*d*):**	5%
**Design effect (for cluster surveys-*DEFF*):**	1
Sample Size(n) for Various Confidence Levels	
**Confidence Level (%)**	**Sample Size**
90%	225
95%	297
99%	440
**Equation**	
Sample size *n* = [DEFF*Np(1-p)]/ [(d^**2**^**/Z**^**2**^_1-α/2_*(N-1)+p*(1-p)]	

### Procedure (data collection)

#### Design of the questionnaire

The questionnaire was prepared based on information from the International Classification of Functioning, Disability and Health (ICF) [29], MACI rehabilitation professionals’ and medical experts’ perspectives and the Cognitive Failures Questionnaire (CFQ). The complete questionnaire contained three different parts, 1) demographic questions, 2) CFQ questionnaire and 3) e-services questionnaire (S1 File).

Information letters containing the purpose of the study, the procedures for data collection, and ethical considerations such as confidentiality and anonymity as well as informed consent forms were provided to all participants. The demographic questionnaire contained questions regarding participants’ age, sex, education, career situation, type and period of the brain injury.

The CFQ is a 25 items self-assessment questionnaire that has been designed to measure difficulties/failures in performance of everyday tasks that occur to people at least occasionally [30]. In the initial validation of the scale, Broadbent et al. proposed that the CFQ measures a general factor of cognitive failure including perception, memory, and motor function. However, several researchers have argued that the CFQ measures several factors (e.g., 1 factor, 2 factors, 3 factors, 5 factors) and confirmatory and exploratory factor analysis was used to observe different items which have similar patterns [30–33].

The questionnaire has been translated into different languages and been used in various countries with diverse occupational groups [31–34]. The Swedish version of the CFQ was used in this survey. It contains questions such as “Do you forget your appointments?”, “lose your temper and regret it” or “fail to notice something although it is there”. As an answer to each item, participants use a 5-point Likert-scale (i.e., 0 = never, 4 = always) and total scores can range from 0 to 100. Since cognitive lapses occur frequently in the general population, there is a need to differentiate healthy behavior from pathological. The summarized comparison scores are available for different populations [35]. For the present study a percentile analysis was performed on the total CFQ scores (TCFQS) to identify those reporting a large number of cognitive problems and separate them from persons with medium and lower rates.

To assess participants’ use of and experiences with e-services and social media, no adequate survey instrument regarding people with MACI and experiences with e-services was found in the literature. Therefore a new questionnaire was developed based on the most common and important problems for persons with MACI which have been identified on relevant chapters of ICF [11]. For this questionnaire, a few different ICT questionnaires such as experience with e-services and ICT as social support were reviewed and 12 items (navigation, alarm, memory, watching, listening, playing, reading, writing, communication, banking, health promotion apps and services, seeking health information) were identified and cross validated with the ICF classification and questions were presented as different examples for each item in the survey. Table 2 shows the examples that were used in the survey to specify every item for the participants. Participants were also asked if they use computers, mobile phones/smartphones or tablets. Then the use of these devices for different activities related to ICF items such as reading, finding the way, cognitive support, alerts etc. was studied. Finally, open-ended questions were posed about participants’ preferences and positive/negative aspects of using e-services in the daily life. In order to understand the social aspect of these e-services, a few questions about specific social group memberships or e-services were added to the questionnaire. An advisory group of MACI and ICT experts was asked to assist the authors in phrasing the questions in an appropriate manner.

**Table 2:** Examples presented in the survey for e-services items.

e-services	Proposed examples
Navigation	GPS
Alarm	Reminders to sleep and/or wake up, reminders to take medicine
Memory	Digital notes, calendars, journals, to do lists, shopping lists, contact lists
Watching	Such as videos from YouTube or similar services …
Listening	Audio books, music from Spotify, internet radios or …
Playing	Through various computer games or consuls
Reading	eBooks
Writing	Taking notes or …
Communication	Via email, chat and Facebook. Find out the facts through Google, news aggregators, subscribe to newsletters, etc.
Banking	Economy management, count and make purchases such. E.g., save your receipt, calculator and pay and make transfers via Internet banking, paying bills, wallet
Health- promotion apps and services	Such as measuring blood pressure, weight, pulse, sleep or diet apps
Seeking health information	Seeking health information

In April 2014, the questionnaire was pilot tested with two persons with MACI and revised according to their comments. By the end of May 2014, questionnaires were sent out to 600 members of Hjärnkraft. By the end of December 2014, 226 replies were received and a follow up reminder was sent including an additional question asking participants if they are not interested in the study or not able to participate in the study due to physical or mental disabilities. By the end of January 2015, 171 more participants replied.

#### Data analysis

Firstly, “SPSS” version 23.0.0.0 statistical software package was used for a descriptive analysis of study participants and to determine normality for the data collected from multiple choice and close-ended questions. For continuous variables such as age and TCFQS, normal distribution was verified by the Shapiro–Wilk normality test and the 1-sample Kolmogorov–Smirnov test. To determine significant differences within different demographic data, depending on normality and type of independent variables (interval, ordinal and nominal with two or more than two levels), different statistical tests were applied. For normally distributed variables the Welch 2-sample t test and for non-normally distributed variables the Kruskal–Wallis rank sum test Mann-Whitney U or the Wilcoxon rank sum test with continuity correction were used. Test results in which the p-value was smaller than the type 1 error rate of 0.05 were declared significant. In addition, the answers to open-ended questions regarding the participants’ positive and negative sentiments towards using e-services were worked through and analyzed to identify major trends.

## Results

### Characteristics of the participants

During the enrolment period, 397 out of 600 members (66%) replied to the invitation and 282 members (47%) were interested and able to participate in the survey. In addition to survey responses, other participants called the data manager to describe their non-engagement reasons. Table 3 shows the participation rate for the study.

**Table 3:** Participation rate.

Participation Type	Number of participants
	*n*(%)
Interested	282(47)
Not Interested	41(7)
Not able due to health condition	63(11)
Unknown Address/Deceased	11(2)

Considering the estimated population size (about 1300 registered members), the confidence level for this study was close to 95% (Table 1) which showed that the sample size was large enough to reflect upon the estimated population.

### Cognitive failure questionnaire

The mean of TCFQS for the participants was 44.9 ± 18.2. The mean of TCFQS was not significantly different between participants who used and those who did not use e-services. Considering different demographic characteristics, such as sex, gender, education, employment and the type and period of brain damage, mean TCFQS varied between 40–50 points for each category. The “R” statistical software was used to test the factor structure of the CFQ using confirmatory and exploratory factor analysis. The confirmatory analysis did not support any factors proposed by Wallac et al. and Rast et al. and the results of exploratory factor analysis supported the one factor solution [34,36]. As mentioned in the methods section, the participants of this study were divided into different groups based on their TCFQS’s percentile pattern to show a better understanding of the TCFQS results and used e-services. Based on the analysis a score of 31 points and 58 points were taken as 25^th^ and 75^th^ percentile for TCFQS values to divide participants into three groups (Table 4).

**Table 4:** Percentile analysis of TCFQS for all participants.

		Percentiles						
		5	10	25	50	75	90	95
**Weighted Average(Definition 1)**	**TCFQS**	17.00	22.30	31.00	44.00	58.25	70.00	76.00
**Tukey's Hinges**	**TCFQS**			31.00	44.00	58.00		

Table 5 shows the groups division after the percentile analysis.

**Table 5:** Group division based on percentile analysis.

TCFQS	Number of participants	TCFQS
	*n*(%)	mean±SD
**TCFQS <31 (Low)**	66(23)	21.2±7.8
**31< = TCFQS> = 58 (Medium)**	146(52)	44.2±7.6
**TCFQS >58 (High)**	70(25)	68.8±7.4

### Participants’ demographic characteristics

Participants’ demographic characteristics are displayed in Table 6. Most participants were 40 to 80 years old, with the largest group (45%) being between 50 to 65 years old. The lowest percentage of participants came from the 13 to 29 year age group. Females in this study had significantly higher TCFQS compared to male participants (*p*<0.05). The majority had high school education and about one-third had a college/university degree but only 21% were employed. For most of the participants (65%), the brain damage incident happened more than 10 years ago and the main cause was most often (40%) traumatic brain injuries such as traffic accidents or falls (20% left this field blank).

**Table 6:** Participants’ demographic characteristics.

Demographic characteristics	Responses	Number of participants	TCFQS	*P-value*
		*n*(%)	Mean±SD	
**Sex**	Male	157(56)	41±18	**0.000**
	Female	125(44)	50±18	
**Age**	13–29	21(7)	46±20	**0.592**
	30–49	82(29)	42±19	
	50–65	126(45)	47±18	
	66–81	53(19)	44±16	
**Education**	Elementary	61(22)	47±21	**0.844**
	High school	118(42)	45±17	
	College/University	91(32)	45±18	
	No Answer	12(4)	-	
**Employment**	Employed	58(21)	42±18	**>0.05 by different tests**
	Studying	11(4)	40±21	
	Sick leave	24(9)	44±20	
	Sickness compensation	72(26)	50±18	
	Unemployed	7(2)	42±14	
	Retiree	108(38)	44±18	
	No Answer	2(1)	-	
**Period of brain damage**	Less than 2 years	13(5)	41±22	**>0.05 by different tests**
	Between 2 to 5 years	33(12)	42±21	
	Between 5 to 10 years	53(19)	48±16	
	More than 10 years	182(65)	44±18	
	No Answer	1(0)	-	
**Type of brain damage**	TBI	113(40)	45±20	**>0.05 by different tests**
	Anoxia	2(1)	52±19	
	Stroke	58(21)	47±19	
	Tumor	17(6)	50±12	
	Encephalitis	11(4)	47±19	
	Other / Unclear	25(9)	41±18	
	No Answer	56(20)	-	

Nearly two-third of the participants (66%) had a personal computer but only 23% owned an electronic tablet. Among 81% who owned mobile phones, half of them (41%) claimed to have a smartphone and they had a significantly higher TCFQS (*p*<0.05) (Table 7).

**Table 7:** Participants’ electronic device ownership.

Electronic device	Responses	Number of participants	TCFQS	*p*
		*n*(%)	Mean±SD	
**Personal Computers ownership**	Yes	186(66)	46±17	**0.437**
	No	93(33)	44±20	
	No Answer	3(1)	-	
**Mobile phones ownership**	Yes	228(81)	46±17	**0.440**
	No	52(18)	43±23	
	No Answer	2(1)	-	
**Smartphones ownership**	Yes	115(41)	49±18	**0.013**
	No	101(36)	43±16	
	didn't know	5(2)	38±15	
	No Answer	61(22)	-	
**Tablets ownership**	Yes	64(23)	43±18	**0.332**
	No	215(76)	46±18	
	No Answer	3(1)	-	

**Fig 1:**
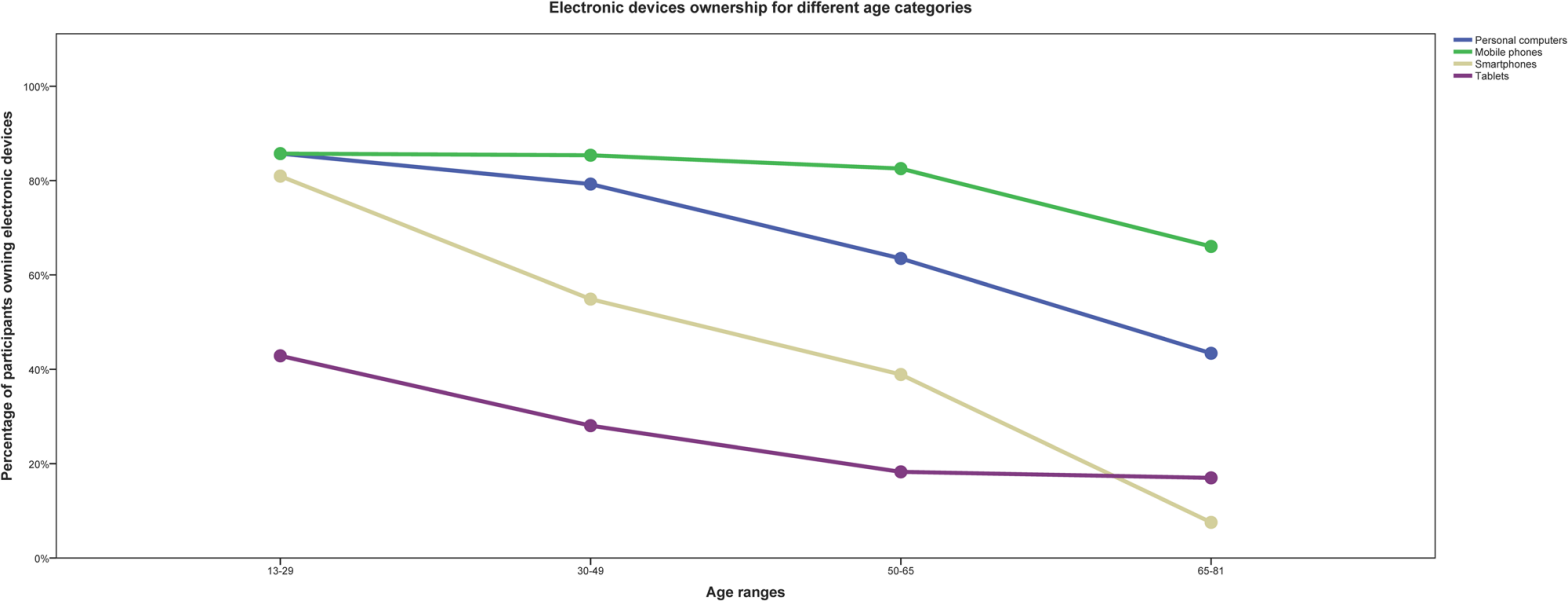
Electronic device ownership for different age categories.

Except for differences in TCFQS between men and women and in smartphone ownership, other analyses based on demographical differences did not show any significance whatsoever. Disregarding participants’ cognitive failure rate, most of them owned mobile phones and personal computers in all age categories. Smartphones and electronic tablets were more popular among younger participants. Fig 1 shows device ownerships for different age categories.

### E-services

Based on the results of participants’ responses to 12-items e-services, about 89% (250 of 282 members) of the participants were using some kind of e-services on their personal computers, mobiles or tablets. Most of the participants used e-services for Communication and Banking. Among all suggested e-services items, reading and health promotion services (Apps) were the least popular ones. The average age for the participants who used e-services was 43 years old. Participants who were using reminders, such as alarm or memory, listening and communication services had significantly higher TCFQS compared to others (*p*<0.05) (Table 8).

**Table 8:** Participants’ responses to e-services questionnaire.

e-services	Responses	Number of participants	TCFQS	*p*
		*n*(%)	Mean±SD	
**Navigation**	Yes	85(30)	46±18	**0.499**
	No	197(70)	44±18	
**Alarm**	Yes	95(34)	51±17	**0.000**
	No	187(66)	41±18	
**Memory**	Yes	87(31)	50±18	**0.002**
	No	195(69)	43±18	
**Watching**	Yes	68(24)	46±18	**0.691**
	No	214(76)	45±18	
**Listening**	Yes	77(27)	50±18	**0.006**
	No	205(73)	43±18	
**Playing**	Yes	75(27)	46±20	**0.795**
	No	207(73)	45±18	
**Reading**	Yes	14(5)	47±22	**0.788**
	No	268(95)	45±18	
**Writing**	Yes	93(33)	47±19	**0.124**
	No	189(67)	44±18	
**Communication**	Yes	165(59)	47±17	**0.006**
	No	117(41)	41±19	
**Banking**	Yes	110(39)	44±18	**0.373**
	No	172(61)	46±19	
**Health promotion**	Yes	13(5)	53±20	**0.124**
	No	269(95)	45±18	
**Seeking health information**	Yes	78(28)	47±18	**0.201**
	No	204(72)	44±18	

**Fig 2:**
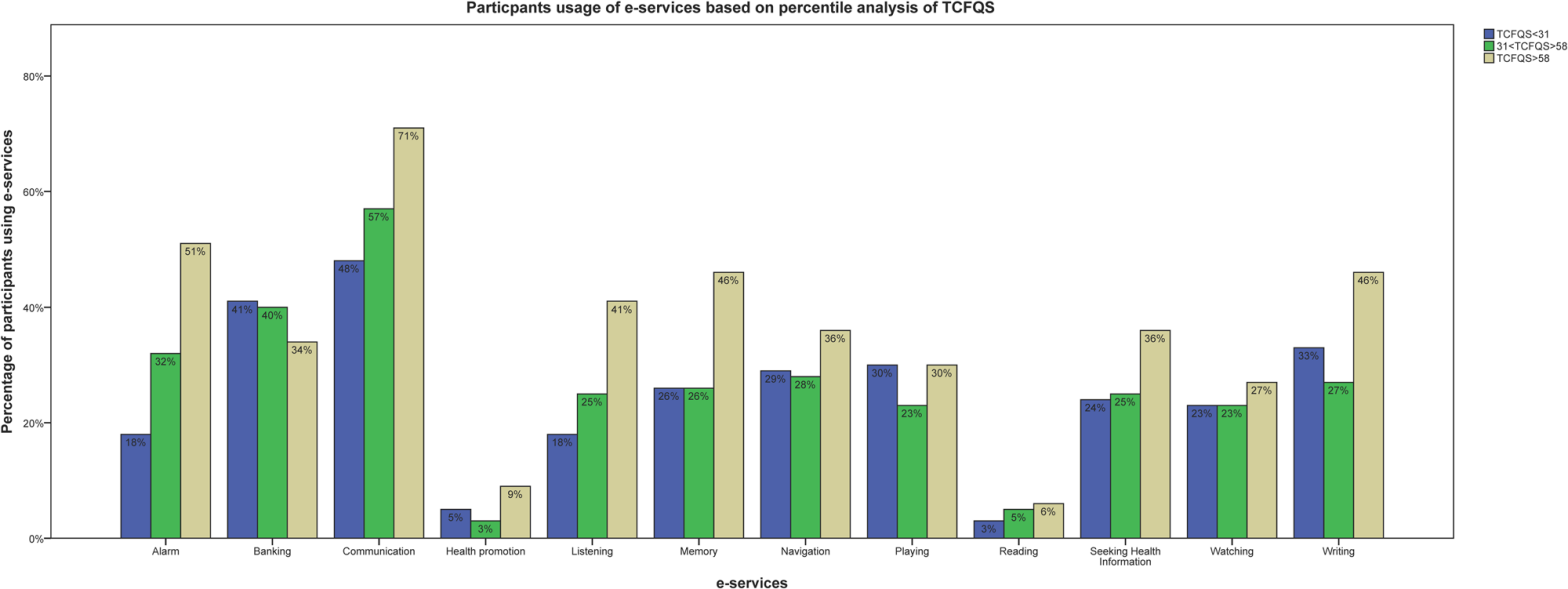
Participants use of e-services comparison based on total CFQ score.

The results showed that participants with higher TCFQS were using e-services more than participants with lower and medium TCFQS did. However, the percentage of participants with lower CFQ scores who used banking services was slightly higher (Fig 2).

Based on the statistical analysis and regarding the participants’ sex, females, independent of cognitive level were using more e-services in almost every category than males. The sole exception was multi-media services (watching and listening), which were more popular among male participants with higher TCFQS. Males with low TCFQS were the least frequent users of e-services in almost all categories. Considering the participants’ education, watching and playing services were most popular among participants with high school education and high TCFQS. However, participants with college/university education and higher TCFQS were using the e-services more than the other groups.

The participants’ responses to open-ended questions were mostly one word or very short sentences. Table 9 shows a few quotes which were taken from more elaborated responses. Most participants had positive attitudes towards using e-services and generally, e-services were used for different types of support mainly as a mean for communication aimed at different matters such as socializing and having a reliable connection for help and emergencies. Social media was a unique experience for many participants to find new friends and cheaper than phone for communicating with relatives. Generally, e-services and the Internet were considered a great source of information in addition to providing means of education, training and entertainment. According to the participants’ responses, availability, mobility, flexibility and rapidity were the most positive aspects of e-services and they considered mobile phones in general being very useful. The main negative aspects for participants of this study relate to behavioral influence, difficult design and technical downsides. Issues such as intrusion of privacy and risk for addiction were their main concerns. On the other hand, they were unsatisfied by software and hardware problems such as the Internet or electronic devices’ speed, battery life, unwanted pop-ups, advertisements and viruses while using e-services.

**Table 9:** A few quotes taken from participants’ responses to open-ended questions.

Quotes
“Being able to text/call my husband to let him know where I am and how I feel.”
“Free information with no social effort.”
"Trying to solve Bingo game on Saturdays."
“I use the camera as a reminder.”
“Audio recording for repeating meetings, etc. Remembering my own via audio recording”
“Works as my external brain.”
“Read about others who have had a brain injury”
“A gymnastic tool for my brain.”
“All reminders you carry in your pocket”
"Easy to get contacted by others.”
“You can take it at your own pace and others can take it at their own Pace."
“It is the cheapest phone that you have it with you”
“Can do things even when I was resting on the bed (smartphone, mobile).”
“Security of being able SMS/call my husband to let him know where I am and how I feel”
“Internet is cheaper than phone.”
"I can be in Facebook and listening to music"
“All in one, numerous help functions in one equipment.”
“I have a freedom to do what I want”
“Mobile is smooth, not as distracting and annoying as a computer.”
"I'm impulsive and can give angry response to someone in a chat"
"I quickly get tired"
"Finding something is difficult and waiting for the page to load"
"It’s hard to find what you came for (a lot of advertisements or other suggestions in the webpages that grab you attention)"
"I am not skilled. I encounter many problems in both the computer and the mobile. For example I'm doing an incredible number of typing errors on your PC and have difficulty remembering steps in a process of both."
“The risk with all these getting fooled by people who want money and different information”

#### Social network membership and preferred e-services

In response to questions regarding social network memberships and the Internet services, participants mentioned different e-services and websites (Table 10).

**Table 10:** Preferred e-services according to participants’ responses.

Type	e-service
**Communications**	Skype
**General aids**	Spelling aid programs
	Text readers
**Games and entertainments**	Big Brain (Wii)
	Lumosity
	http://www.gamescampus.com
	http://www.svenskspel.se
	http://www.youtube.com
**Information sources**	http://www.hitta.se
	Google
**Education and training**	http://www.gleerups.se
	http://www.endomondo.com
	http://www.cogmed.com
	http://www.comai.se
**Patient associations**	http://www.fusig.se
	http://www.hjarnkraft.nu
	http://www.slussa.se
	http://www.smil.se
**Social networks**	http://www.stayfriends.se
	Facebook
	Instagram
	Twitter

Among the social media applications (or services), Facebook was the most popular one (26%) followed by Twitter (2%) and Instagram (2%). The average age for Facebook users was 46 (Table 11).

**Table 11:** Age distribution for Facebook users.

Age	Number of participants	%	TCFQS mean±SD
**13–29**	10	4	49±15
**30–49**	29	10	46±20
**50–65**	30	11	47±17
**66–81**	3	1	43±31

## Discussion

The results of this survey concerning persons with mild and moderate acquired brain injuries showed that a large proportion of these persons use e-services and show a positive attitude towards using e-services to help them achieve a more self-regulating and independent life. However, we do lack data about the members who did not participate in the study which is a limitation.

Previous work revealed only a limited number of studies in this area, showing that persons with more limited impairment are an understudied group among the research community [11]. Also when it comes to use of e-services, literature reviews showed that research and technology development focused more on single problems (one specific device/service) for persons with more severe cognitive problems [37]. Our results indicate that persons with limited cognitive impairment frequently use commercially available e-services and have various needs and interests in this area. The present study also holds several useful implications and ideas for future research.

### Cognitive level of the participants

Cognitive level was estimated by self-ratings. As indicated in Table 4 there was a wide range of responses. The mean for TCFQS was very close to what Wallace et al. [34] (43 ± 18) and Matthews et al. [32] (45 ± 10) have reported with different study populations such as healthy university students. However, the mean was higher than healthy controls reported by Rijsbergen et al. [38] (29.2±10.3) which indicated cognitive failure for the participants of this study. A conspicuous number of participants reported a limited number of problems, which, considering the history of verified brain damage in the group, might reflect a number of factors, such as lack of insight [39].

The obtained self-reported TCFQS did not correlate with either age or the type/period of brain damage for the participants of this study. This was due to the variety of participants in terms of type/ period of brain damage. However, the TCFQS seem to be helpful to show that these persons experience cognitive problems and measure a general liability to cognitive failure.

### E-services

Regarding the diversity of e-services used by the participants, we found that the ICF classification was a valuable tool for exploring the functionality of e-services and it provided a good insight about different categories of e-services to support this group. Based on the participants’ responses, all of their current and imaginary e-services fit into sub-sets of frequent and crucial problems for MACI patients as described by Eghdam et al. [11].

Despite participants’ cognitive failure, the use of e-services in general among the study population (89%) was comparable to the Swedish general population (93%) [18]. Considering the responses, participants were interested in using e-services of almost every category. The results showed that personal computers, smart phones and tablets have the potential to be used more by persons with MACI.

Generally, ICT equipment was considered as a support and a reliable device to trust in different situations. The results showed that the Internet is a great source of general and health information, simple and diverse to interact, and a manner to use for education and training for this group. Undoubtedly, based on participants’ answers to open-ended questions, seeking health information and freedom/opportunity in asking questions regarding their health, played a great role in their usage of the Internet. However, using some aspects of e-services, privacy and security issues in addition to behavioral influence and resistance feelings were problematic for some of the participants and as Cottet et al. indicated, the primary characteristics of online health information seekers must be investigated to better recognize their needs and to enhance the health information quality and availability for this specific group [40]. This might be a result of an individual’s innate conservatism and lack of felt need as factors influencing resistance to ICT that exist amongst the general public and can be resolved by accurate guidance in a precise context [41].

#### Communication and social media

In our previous study we have noticed that the ICF category “social interaction and relationships with families and friends” contains important issues for persons with MACI [11]. Therefore, one of our concerns in the planning phase of this study was social relationships with families and friends and generally communication aspects of e-services.

Based on the findings, the most popular and essential category for the participants was communication services such as email, chat, newsletters, social media and etcetera. Comparing the results of using social media like Facebook, this group does not use social media as frequently as the general public [18]. However, the proportion of participants who use Facebook is rather quite noteworthy despite the reported cognitive problems. Although many participants were very interested in social media, they were annoyed by advertisements and the Internet speed in general. It would be beneficial to test the idea of ad-block softwares/filters to see if it makes the use of communication services easier for them. In general, the communication/social aspect of e-services might be a priority for future research to investigate more for this specific group. Furthermore, services such as shopping, banking and booking facilities were relatively popular among persons with limited cognitive impairment, which indicates e-commerce features of e-services have to be considered for more investigation as well.

#### E-service and aging

Most of the participants in this sample were middle aged and most of them had their brain injuries more than 10 years ago (65%). They use e-services while trying to reclaim and maintain earlier lifestyle, everyday activities and habits. Bearing in mind that e-services are quite new in human kind’s life and the struggle to learn to use a new device, the power and influence of ICT is impressive.

The results showed that usage of electronic devices decreased by age but surprisingly older participants were using electronic tablets approximately the same as other age groups. This shows the great potential for electronic tablets to become a great e-services channel for older adults with MACI.

#### Experience with e-services

More than three quarter of the participants were using various e-services. Bearing in mind the average age and TCFQSs, these services/tools seems to be part of their daily lives, even though they are not specifically designed for them. The proportion of participants interested in other aspects of e-services such as gaming, music services, writing and seeking health information rather than just communication and banking, was also significantly high (more than 25% of the participants). Considering that, these services are not customized for this group; the results are impressive that persons with mild to moderate cognitive difficulties without longer previous experience as in middle aged and older adults, use such services. Further studies are needed to explore how knowledge about the use of e-services is acquired in this group.

Among all the proposed categories, very few participants used self-care health services (apps) and readers (e-readers) (Fig 2). The results specified that persons with MACI are able to use e-services on their own. However, the answers to open-ended questions showed that participants had difficulties with distraction, concentration, tiredness and information overload when using e-services. Certainly, technology difficulty was one of the issues for them but there was not any insight if they were using old devices or whether the difficulty was due to their disabilities. This could be useful for future studies to perform an evaluation of the new technology with this population to gain a better understanding of the problematic areas. However, since the available technology is imperfect, some of their concerns such as constant updates, battery life and fragileness are inevitable at this stage. In addition, not using some of the e-services might be due to different reasons such as not being familiar with the existence of these tools or not being provided with well-designed ones. This may possibly be followed in future research to see whether the participants could be encouraged to use self-care health services (apps) by providing more information about them through newsletters from the patient associations or any other information channels or if the problem needs to be addressed by designing special tools for people with special needs regarding self-care services. The commercially available technical support is not adequate for this group and their needs within the health-care system are ignored.

Participants with higher TCFQS, i.e. more cognitive problems, were using most of the e-services; more than other participants. Considering the mentioned difficulties in acquiring the technology, learning and using e-services, this fact indicates that technology is a good support in general for people with more cognitive problems. Hofer suggests that generally when people face cognitive challenges, they prioritize the optimal and trustworthy resources rather than investing in learning new things or working with new equipment [42]. They maintain their abilities to the current level by using the available resources without putting more effort in using a new one. In this case, the available e-services might be that resource for these people. Rehabilitation resources are more restricted in Sweden for persons with MACI, thus one hypothesis would be that people with MACI receive less external support in acquiring new skills after their brain injury. Also occupational therapists need to improve their skills in training patients to use e-services [43].

Certainly, ICT in general can help persons with MACI in managing their health, easily and safely. Moreover, most of them with basic knowledge of ICT were interested in testing and using the tools in further studies (43%). Their interest indicates that even though they might not have used the services, they have a very positive attitude towards innovative technologies.

To the best of our knowledge, this study is one of the first to show persons with mild acquired cognitive impairment’s usage of e-services. However, it would be beneficial for the health informatics scientific community to replicate the study with similar participants as the finding has to be confirmed. In addition, it is imperative to see how the usage of existing e-services supports and improves self-management of persons with MACI.

### Limitations

One limitation of the present study was the reliance on self-reported cognitive problems when investigating the use of e-services in this group, which may be confounded by the very same factor it aims to measure. Since the participants of this study were only members of one Swedish association, the results cannot be generalized for other populations and countries, which may not be equally familiar with e-services. In addition, the selection of participants may have introduced a selection bias since there is a lack of data about members who did not participate in the study. Considering the participants and the results, other than what has been presented here, we cannot draw any conclusions of significance because the participants’ cognitive failure was due to different types of injuries in addition to the age factor. In addition, the details about the frequency of using e-services in participants’ daily lives and financial support for affording one are missing in the results of this survey.

## Conclusions

Our survey study of 282 persons with MACI demonstrated that the majority of participants were using personal computers and mobile devices mostly as communication and banking aides and they have a positive attitude towards using e-services.

The results showed that areas such as navigation, alarms, memory, video and music service, writing, banking, seeking health information and specifically social interaction services are the most important aspects of this technology for this group. However, further studies are needed on utilizing these identified aspects for this specific group to support them with their chronic condition. Similarly, further efforts are needed to popularize persons with MACI among the research community as a group with special needs and necessities toward using e-services. It may be interesting to explore the usage of the Internet and social media more on an individual level, further exploring the relationship between cognitive function and the use of e-services. In addition, it would be motivating to explore the long-term impact of information exchange between persons, families, and caregivers, and the long-term effects of using such technologies by follow-ups.

## Supporting Information

S1 File The ICT-CFQ questionnaire. (DOCX)
