# Supplementary material for: How Do Persons with Mild Acquired Cognitive Impairment Use Information and Communication Technology and E-Services? Results from a Swedish National Survey
Source: PLoS One. 2016 Jul 18;11(7):e0159362. doi: 10.1371/journal.pone.0159362 (PMC4948839; doi:10.1371/journal.pone.0159362)
Supplement: S1 File — (DOCX) [file pone.0159362.s001.docx]

# Questionnaire 1

**The Cognitive Failures Questionnaire** The following questions are about minor mistakes which everyone makes from time to time and some perhaps more often than others. We want to know how often these things have happened to you in the past 6 months. Please select the option that fits your answer best.

|  |  | Very  often | Quite often | Occasion ally | Very  rarely | Never |
| --- | --- | --- | --- | --- | --- | --- |
| 1. | Do you read something and find you haven’t been thinking about it and must read it again? |  |  |  |  |  |
| 2. | Do you find you forget why you went from one part of the house to the other? |  |  |  |  |  |
| 3. | Do you fail to notice signposts on the road? |  |  |  |  |  |
| 4. | Do you find you confuse right and left when giving directions? |  |  |  |  |  |
| 5. | Do you bump into people? |  |  |  |  |  |
| 6. | Do you find you forget whether you’ve turned off a light or a fire or locked the door? |  |  |  |  |  |
| 7. | Do you fail to listen to people’s names when you are meeting them? |  |  |  |  |  |
| 8. | Do you say something and realize afterwards that it might be taken as insulting? |  |  |  |  |  |
| 9. | Do you fail to hear people speaking to you when you are doing something else? |  |  |  |  |  |
| 10. | Do you lose your temper and regret it? |  |  |  |  |  |
| 11. | Do you leave important letters unanswered for days? |  |  |  |  |  |
| 12. | Do you find you forget which way to turn on a road you know well but rarely use? |  |  |  |  |  |
| 13. | Do you fail to see what you want in a supermarket (although it’s there)? |  |  |  |  |  |
| 14. | Do you find yourself suddenly wondering whether you’ve used a word correctly? |  |  |  |  |  |
| 15. | Do you have trouble making up your mind? |  |  |  |  |  |
| 16. | Do you find you forget appointments? |  |  |  |  |  |
| 17. | Do you forget where you put something like a newspaper or a book? |  |  |  |  |  |
| 18. | Do you find you accidentally throw away the thing you want and keep what you meant to throw away – as in the example of throwing away the matchbox and putting the used match in your pocket? |  |  |  |  |  |
| 19. | Do you daydream when you ought to be listening to something? |  |  |  |  |  |
| 20. | Do you find you forget people’s names? |  |  |  |  |  |
| 21. | Do you start doing one thing at home and get distracted into doing something else (unintentionally)? |  |  |  |  |  |
| 22. | Do you find you can’t quite remember something although it’s “on the tip of your tongue”? |  |  |  |  |  |
| 23. | Do you find you forget what you came to the shops to buy? |  |  |  |  |  |
| 24. | Do you drop things? |  |  |  |  |  |
| 25. | Do you find you can’t think of anything to say? |  |  |  |  |  |

# Questionnaire 2

**How do you use computer, mobile phone / smartphone, tablet and Internet.**

1. Age:……..
2. Sex:

Male Female

1. What is your current job? .......................
2. What is your education?

Elementary school High school College/University

1. Employment:

Working:

Fulltime job Part time →percentage: ........

- - Studying
  - On sick leave → percentage: ........................
  - Sickness benefit → percentage: ....................
  - Unemplyed
  - Retired (Pensioner)

1. How did you get your brain injury? ....................
2. When did you get your brain injury?
   - Less than 2 years ago
   - Between 2 and 5 years ago
   - Between 2 and 5 years ago
   - More Than 10 years ago
3. Do you use computer regularly? Yes No
4. Do you use mobile phone? Yes No
   - If yes, is it a smartphone? Yes No I don’t know
5. Do you use any tablets? Yes No

***NOTE: If you do not use any of the services mentioned in question 8,9 and 10, the survey ends here and you do not need to go through the rest of the questions. Even if you only use one of them, please continue to complete the survey!***

1. In what way do you use tablet / mobile / computer today? (Please select from the options below):

Finding your ways by using maps (e.g. GPS)

Alarm or reminder - to sleep and / or wake up, reminders to take medications

Memory supports– notes, calendars, journals, to do list, shopping list, contact list, etc ...

- Watching videos (e.g. videos from Youtube or ...)
- Listening (e.g. Audio books, music via Spotify, Internet radios)

Playing games (e.g. through various video/computer games)

- Reading – eBooks

Writing – take notes, upload address lists e.g. of friends and etc…

Communication – Via email, chat and Facebook. Find out the facts through Google, news aggregators, subscribe to newsletters, etc.

- Economy management, count and make purchases such. E.g., save your receipt, calculator and pay and make transfers via Internet banking, paying bills, wallet
- For your own health (such as measuring blood pressure, weight, pulse, sleep or diet apps)
- Seeking health information
- Others………..........…..

1. Do you use any Internet service that helps you with your forgetfulness, difficulty concentrating, or other cognitive problems? Yes No
   - If yes, please describe which services ….................................................
2. Do you use the Internet to connect with other people with similar cognitive problems?

Yes No

1. Are you a member of any social networking groups on the Internet? Yes No
   - If yes, which ones? ...................................................................................
2. What are the most positive aspects of

- Using the tablet / smartphone / mobile / computer................................................
- How about Internet?...............................................................................................

1. What are the most negative aspects of

- Using the tablet / smartphone / mobile / computer................................................
- How about Internet?...............................................................................................

1. Are you interested in participating in the futhur research about computerized / electronic supports for people with cognitive difficulties? Yes No

If you are interested, we invite you to a meeting where we talk further about your participation and the procedure. For us to be able to invite you to the meeting, we need your contact information;

Name: ……………………………………...

Telephone: …………………………………....

Mobile: ………………………… ……….

email: ……………………………….
